# Early Mixed Farming of Millet and Rice 7800 Years Ago in the Middle Yellow River Region, China

**DOI:** 10.1371/journal.pone.0052146

**Published:** 2012-12-17

**Authors:** Jianping Zhang, Houyuan Lu, Wanfa Gu, Naiqin Wu, Kunshu Zhou, Yayi Hu, Yingjun Xin, Can Wang

**Affiliations:** 1 Key Laboratory of Cenozoic Geology and Environment, Institute of Geology and Geophysics, Chinese Academy of Sciences, Beijing, China; 2 Zhengzhou Provincial Cultural Relics and Archaeology Research Institute, Zhengzhou, China; 3 University of Chinese Academy of Sciences, Beijing, China; Ben-Gurion University, Israel

## Abstract

The Peiligang Culture (9000-7000 cal. yr BP) in the Middle Yellow River region, North China, has long been considered representative of millet farming. It is still unclear, however, if broomcorn millet or foxtail millet was the first species domesticated during the Peiligang Culture. Furthermore, it is also unknown whether millet was cultivated singly or together with rice at the same period. In this study, phytolith analysis of samples from the Tanghu archaeological site reveals early crop information in the Middle Yellow River region, China. Our results show that broomcorn millet was the early dry farming species in the Peiligang Culture at 7800 cal. yr BP, while rice cultivation took place from 7800 to 4500 cal. yr BP. Our data provide new evidence of broomcorn millet and rice mixed farming at 7800 cal. yr BP in the Middle Yellow River region, which has implications for understanding the domestication process of the two crops, and the formation and continuance of the Ancient Yellow River Civilization.

## Introduction

**Figure 1:**
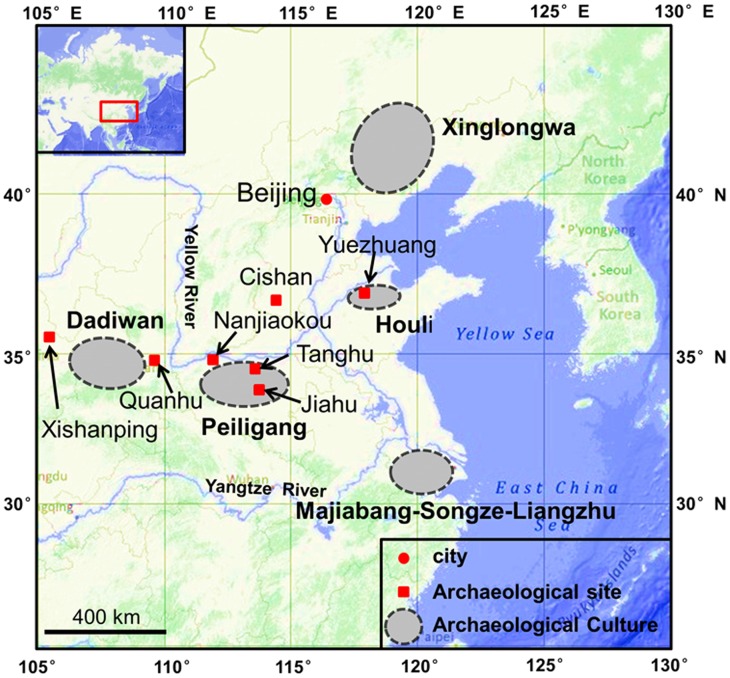
Archaeological sites and related cultures mentioned in the article. The map cited from http://maps.ngdc.noaa.gov/viewers/bathymetry/.

It is generally believed that the Peiligang Culture (*c*. 9000-7000 cal. yr BP) represents a key cultural transition complex from a mobile hunter-gatherer society to a dry farming-based Neolithic culture in the Middle Yellow River region [1]–[3] (Figure 1). Crop remains from the Peiligang Culture have rarely been found before [4]–[6], however, thus several critical archaeobotanical questions related to cultivated crop assemblages are still unresolved. For example, because millet is an agronomic, not taxonomic, group with several genera, 1) what genus and species of millet, broomcorn (*Panicum miliaceum*) or foxtail (*Setaria italica*), was first cultivated in the Peiligang Culture and 2) whether millet was cultivated singly or together with rice during the Late Peiligang period (8000-7000 cal. yr BP) [4], [7], [8]?

Millet has been considered an important part of the crop assemblage at Peiligang Cultural sites [4], but work to date has only provided limited evidence of cultivated millet. Few carbonized crop grains with neither precise ages nor substantial identification from Late Peiligang Culture sites have been identified as possibly foxtail millet [5], [9]–[11]. Some starch grains on stone tools from Peiligang Cultural sites recently were identified in general terms as millet type [7], [8]. So far, no solid evidence for millet can be used to discuss what kind of millet was first cultivated in the Peiligang Cultural period. Thus the millet domestication process is still unclear.

Rice (*Oryza sativa*) has been regarded as a native crop in the Mid-Lower Yangtze River region of China [12]–[14], whereas its presence in the Yellow River region was considered an introduction, because rice evidence was recently found at the Jiahu (*c*. 9000-7800 cal. yr BP) and Yuezhuang sites (Houli Culture, *c*. 8000-7800 yr BP) in the Mid-Lower Yellow River region [11], [15], [16]. No matter whether or not it is native or introduced, rice evidence is absent after the Jiahu and Yuezhuang periods at Peiligang Culture sites (*c*. 8000-7000 cal. yr BP), yet appears again in the middle Yangshao sites (6000-5500 cal. yr BP) in North China [17], [18]. This 2000-year gap of evidence of rice cultivation in the Middle Yellow River region [4], [17] makes it difficult to understand the domestication process, how domesticated rice spread, and how it was combined with millet in mixed farming at this period.

The mixed farming of millet and rice was a significant event in the process of agricultural development. Due to the fact that either millet or rice, not both, has been found at Neolithic sites in Central-North China [11], questions of where and when mixed farming was established still remain. More archaeobotanical evidence from Neolithic sites, especially Peiligang Culture sites, is needed to resolve these questions.

Evidence of early farming is based primarily on macrofossil remains, which are limited for the time when the Peiligang Culture flourished, mostly due the remarkably dissolution of organic material in loess regions, including the Peiligang Culture sites, dominated by a warm and wet environment. Unlike macrofossils, phytoliths are extremely durable silica replicas of plant cells [19], [20], which are found in preserved in various environments without serious decay, even in extremely hot and wet areas, such as in tropical forests [21]. Phytolith analysis is a useful technique to identify certain genera or species of plants [19], [20], especially for identifying broomcorn millet, foxtail millet, and related wild ancestors [22]–[24]. In this study, phytolith analysis was conducted on samples from a Peiligang Culture site, Tanghu, in the Middle Yellow River region of southern North China. Our analyses provide new data for understanding the cultivation and spread of broomcorn millet and rice, and the mixed farming of the two crops in Peiligang Cultural period.

## Materials and Methods

**Figure 2:**
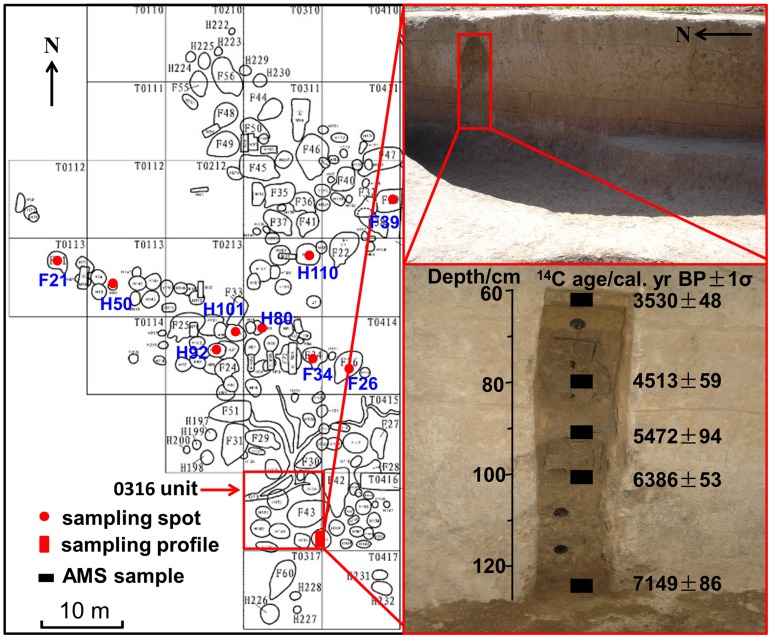
Sampling locations from the Tanghu site. The plan positions of archaeological units is after Xin *et al.* (2010).

The Tanghu site (113°10′43″–113°11′22″ E, 34°7′24″–34°8′20″ N) is located in Henan Province, southern North China (Figure 2). A prehistoric culture, Peiligang Culture, developed there and extended into the Shang and Zhou dynasties [25], [26]. This archaeological site covers *c*. 20 ha and has been considered the largest Peiligang settlement yet discovered [26]. Forty-one semisubterranean round houses (some with double rooms) and 169 pits were found in a 2007 excavation [26]. Unfortunately, no crop remains were found during macrofossil flotation (personal communication with Juzhong Zhang). In this current study, we collected two types of samples for phytolith analysis. One was from the profile of an archaeological layer from excavation Unit 0316, the other was from deposits from pits and houses (Figure 2). All necessary permits were obtained for the described field studies from Zhengzhou Provincial Cultural Relics and Archeology Research Institute.

The deposits from profile (130 cm in thickness) can be divided into five layers from top to bottom according to the structure of the stratigraphy, soil color, and the archaeological remains: 1) 0–22 cm, modern cultivated layer; 2) 22–66 cm, light yellow loess and loose deposits containing ceramic fragments; 3) 66–85 cm, brown soil, dense deposits containing charcoal and ceramic fragments; 4) 85–98 cm, red-brown soil, very dense deposits of red burned soil and charcoal; and 5) below 98 cm, pit deposit (pit H192). We successively collected the samples at 5-cm intervals from the eastern wall of excavation Unit 0316. In total 14 samples were taken from the 60 to 130 cm section, including five AMS dating samples (Figure 2).

Another nine samples were collected from other pits (H110, H101, H92, H80, H50) and houses (F39, F34, F26, F21-4) at the south part of the Tanghu site. These samples were dense deposits of red-brown, and rich in charcoal. Samples H110, H92, F39, and F34 were also used for AMS dating (Figure 2).

Phytolith analysis was conducted on all 23 samples (*c*. 2 g) combining the techniques described by Piperno [27] and Runge *et al*. [28] with slight modifications. The procedure consisted of deflocculating with 5% sodium pyrophosphate (Na_4_P_2_O_7_), then treatment with 30% hydrogen peroxide (H_2_O_2_) and cold 15% hydrochloric acid (HCl), separation using zinc bromide (ZnBr_2_, density 2.35 g cm^3^) heavy liquid, and mounting on a slide using Canada Balsam. Phytolith counting and identification were performed using a Leica microscope with phase-contrast at 400 magnification. In most samples, more than 300 phytoliths were counted. Identification was aided by the use of reference materials [29]–[31] and published keys [9], [19], [32]–[34].

## Results

**Figure 3:**
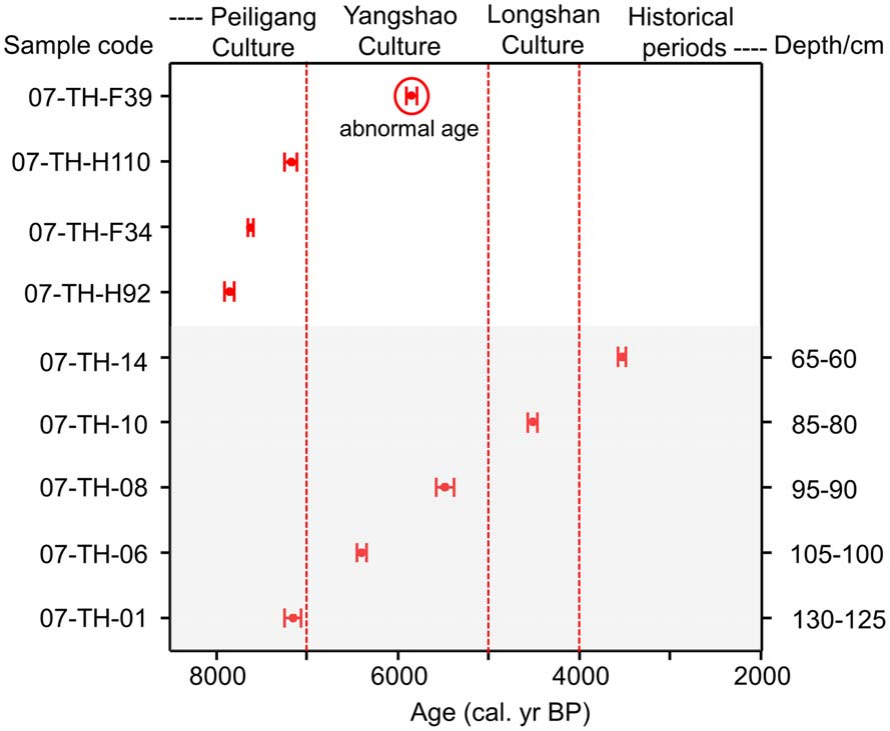
AMS ^14^C ages from the Tanghu site. 07-TH-01, 06, 08, 10, 14 collected from the profile; 07-TH-H92, H110 collected from pits H92 and H110; 07-TH-F34, F39 collected from houses F34 and F39. ^14^C ages made by Guangzhou Institute of Geochemistry, CAS, and the State Key Laboratory of Nuclear Physics and Technology of Peking University. CalPal was used to calibrate the data [59], [60]. Dated materials are paleosoil rich in charcoal.

Ages of AMS dating are given in Figure 3. Nine ^14^C ages, dated using paleosoil rich in charcoal, cover the period from 7840 to 3500 cal. yr BP. Most of ages are consistent with archaeological chronology inferred from archaeological stratigraphy and artifacts [25], [26]. Only one sample (F39, 5843±64 cal. yr BP) dated much later than the age of the Peiligang Culture, so was not used in our study.

**Figure 4:**
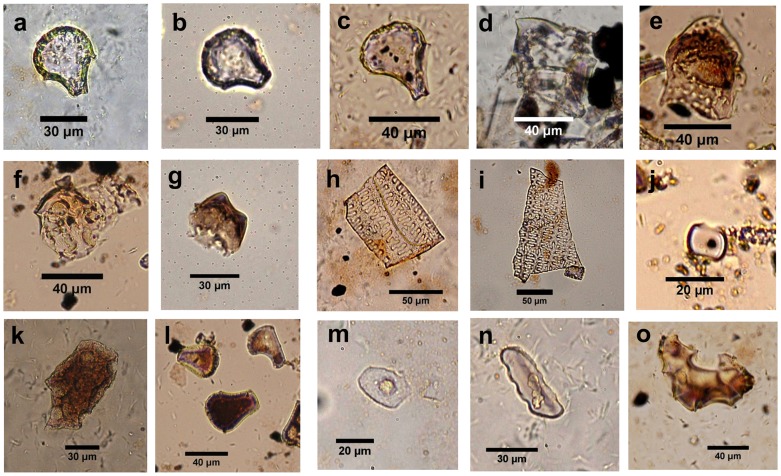
Main phytolith types from the Tanghu site. A–c = rice bulliform; d–g = rice double peaked; h, i = phytoliths from broomcorn millet husk; j = long saddle; k = scutiform-bulliform from reed; l = common bulliform; m = *Cyperus* type; n = trapeziform sinuate (tooth type); o = woody phytolith.

Although some phytoliths appeared partly dissolved, most had distinct features to aid identification. The identification of broomcorn millet phytoliths refers to Lu *et al* [22] and rice double-peaked and bulliform phytoliths refer to the work of Wang and Lu [35], Lu *et al.* [36], Fujiwara [37], and Zhao *et al*. [38], [39]. Finally, a total of 20 phytolith types were identified according to the classification system of Lu *et al*. [40] and three other classifications [33], [35], [41] (Figure 4). Phytolith abundance was expressed as a percentage of all phytoliths counted.

**Figure 5:**
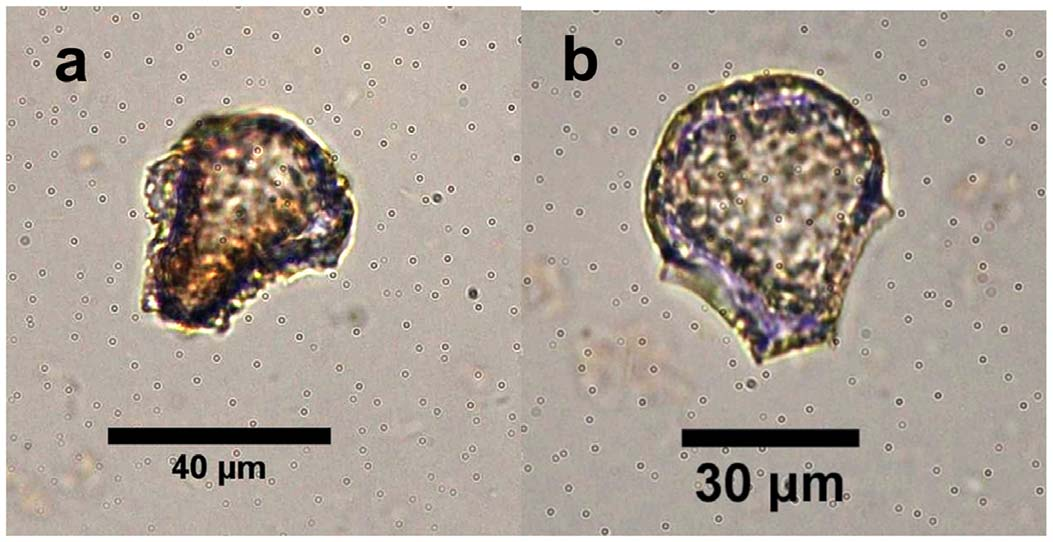
Possible rice bulliforms with dissolved surface from the Tanghu site.

In this study, only rice bulliforms with obvious, uneroded surface features were counted. Fifty-five grains of possible rice bulliforms with dissolved surface were not included in the total samples (Figure 5).

### Phytoliths from the profile

Rice bulliform and double-peaked types were present in the profile at a depth of 105–110 cm (double-peaked; dated older than 6300 cal. yr BP) (Figure 4d), 100–105 cm (bulliform; dated at 6300 cal. yr BP) (Figure 4b), and 85–90 cm (bulliform; dated older than 4500 cal. yr BP) (Figure 4c). No millet phytoliths were found in the profile.

### Phytoliths from Pits and Houses

**Figure 6:**
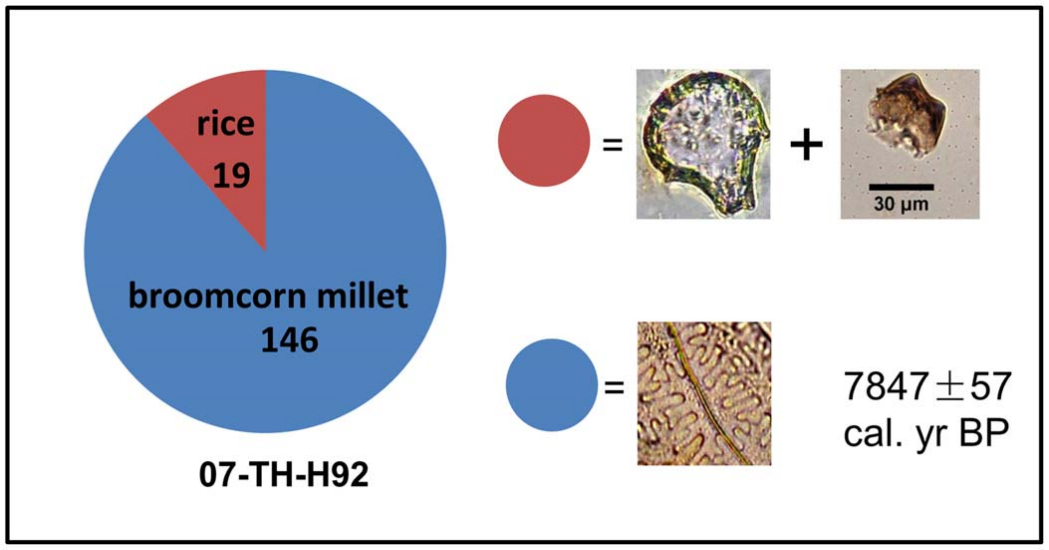
Ratio of phytolith from rice and broomcorn millet in H92 from the Tanghu site.

Sample H92 (7800 cal. yr BP) was from a typical storage pit according to the archaeological excavation [26], [42]. A total of 19 rice phytoliths (including four double-peaks and 15 precise rice bulliforms) (Figure 4a, 4e 4f, and 4g) and 146 pieces of broomcorn millet husks were present in this sample (Figure 4h, 4i). The ratio of rice and millet phytoliths was about 1∶8 (Figure 6). The concentrations of rice and millet phytoliths were, respectively, *c*. 370 phytoliths/g and *c*. 2230 phytoliths/g,.

**Figure 7:**
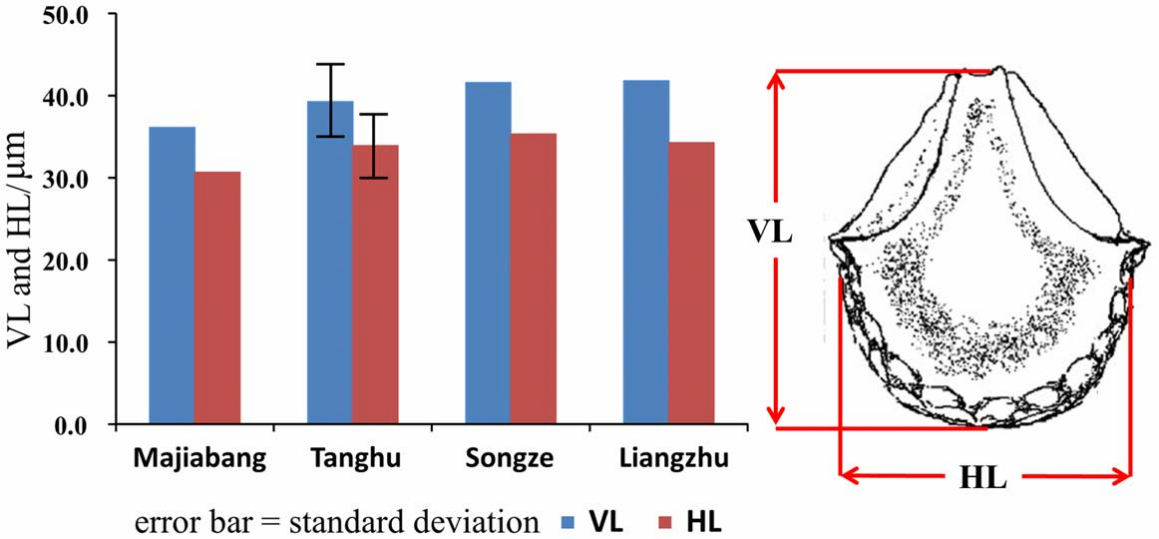
Comparison of morphological characteristics of rice bulliform in difference archaeological sites. The data from the Majiabang, Songze, and Liangzhu Cultures obtained from Zheng *et al.* [34]. The standard deviations of the references are not provided in the original paper. Phytolith portrait is after Fujiwara [37].

Morphological characteristics (vertical and horizontal length) of each individual bulliform were measured following the definition of Zheng *et al* [43]. The average vertical length (VL) of rice bulliforms was 39.3±5.0 µm, horizontal length (HL) was 33.9±4.4 µm, and the ratio of VL/HL was 1.2±0.1 (Figure 7).

Rice bulliform and broomcorn millet phytoliths were found in samples H50 and F21-4 in small quantities (*c.* 0.3%). According to the archaeological stratigraphy and artifacts [25], [26], these two samples were from the Peiligang Cultural period.

In summary, broomcorn millet was present in samples F21-4 and H92, while rice appears in samples H50 and H92, as well as the profile samples at depths of 105, 100, and 85 cm. The temporal coverage of the millet and rice occurrence at the Tanghu site ranges from 7800 to 4500 cal. yr BP. Particularly, rice and millet occurred in the same sample (H92), and tested the same age, 7800 cal. yr BP.

## Discussion

### Dry Farming at the Tanghu Site

Our phytolith results indicated that the millet remains in samples F21-4 and H92 from houses and pits at the Tanghu site were broomcorn millet. These results provided reliable evidence of broomcorn millet cultivation in the Peiligang Culture at 7800 cal. yr BP. Previous studies from Xinglonggou (8000-7500 cal. yr BP) [44], Dadiwan (7800-7300 cal. yr BP) [45], [46], Yuezhuang (7800 cal. yr BP) [11], and Cishan (10,300-8700 cal. yr BP) [47] sites also revealed that broomcorn millet was cultivated first and foxtail millet was barely present during these stages (Figure 1). This means that the former was more significant than the latter in the early stages of food production in North China. Our study also suggests that broomcorn millet was used as a staple food significantly earlier than foxtail millet in the Peiligang Culture. The region that was dominated by broomcorn millet extended to the Middle Yellow River region, southern North China, during the Peiligang Culture period.

### Rice and Millet Mixed Farming in the Tanghu Site

The occurrence of rice at the Jiahu site in southern North China is the earliest presence of rice in North China and can be dated to as early as *c.* 9000-8000 cal. yr BP [15]. After that rice, dated at *c.* 8000 cal. yr BP, is found at the Yuezhuang site north to the Lower Yellow River region [11]. These two findings imply the first northward expansion from the Yangtze River valley of rice cultivation [17]. Previous studies show that no rice was found after 8000 cal. yr BP in Peiligang Cultural sites until it appeared again in the middle Yangshao sites (6000-5500 cal. yr BP) in North China [4], [5], [17], [48]. The absence of rice evidence from 8000 to 6000 cal. yr BP was once considered the southward retreat of rice agriculture to the Yangtze River valley [17]. However, our rice phytoliths from pit H92 and several samples of the profile at the Tanghu site provide new evidence of rice cultivation in the Middle Yellow River region, indicating that rice continued to be cultivated from *c*. 7800 to 4500 cal. yr BP in this region.

The morphology of rice bulliform phytoliths found at the Tanghu site has typical fish-scale-like features on the top and the two lateral protrusions, and more than nine scales arranged along the edge. The latter is considered the distinct morphological feature of cultivated rice [36], [37], although this feature as a distinctive characteristic of cultivated rice needs further validation [17]. Furthermore, morphological characteristics (VL and HL) of rice bulliforms from pits (sample H92) at the Tanghu site (7800 cal. yr BP) (VL: 39.3±5.0 µm, HL:33.9±4.4 µm, N = 15) are bigger than those from the Majiabang Culture (VL: 36.11 µm, HL:30.68 µm) (7000-6000 cal. yr BP), and similar to those from the Songze (VL: 41.58 µm, HL:35.34 µm) (6000-5300 cal. yr BP) and Liangzhu cultures (VL: 41.79 µm, HL:34.28 µm) (5500-4200 cal. yr BP) [34], [49] (Figure 7). Because, on the one hand, the size of rice bulliform phytoliths are associated with individual genes [43], and the trend of becoming larger suggests that rice was utilized or, presumably, cultivated [50], [51]; on the other hand, in mature rice, rice bulliforms are larger than in immature rice [43], so more mature rice in samples can be considered a reflection of a relatively high degree of domestication [14]. Thus, given these quantitative comparisons of bulliform phytoliths, it seems reasonable to speculate that rice was already in the process of domestication during the Tanghu Culture period, similar to the domesticated status from the Songze and Liangzhu cultures in which rice had already been domesticated [14], [17].

Additionally, the morphological comparisons of double-peaked rice husk phytoliths found from the Tanghu site with those in Zhao *et al.* [38] and Itzstein-Davey *et al.* [52] show their resemblance in phytolith morphology with cultivated rice. Furthermore, one representative piece of double-peaked phytolith from H92 was measured and the data (TW:18.8 µm, H:14.7 µm, MW:33.7 µm) are similar to domesticated rice according to Gu *et al.* [53]. Although the quantity of double-peaked rice husk phytoliths is too small to draw any further conclusions, at least its occurrence together with relatively abundant rice bulliform phytoliths indicates that the Tanghu’s residents continued to cultivate rice from *c*.7800 to 4500 cal. yr BP.

The rice phytoliths found in the Tanghu site can also be considered evidence of a northward expansion of rice agriculture from the Middle-Lower reaches of the Yangtze River. Step by step, rice arrived at Nanjiaokou and nearby sites in the north part of Henan Province *c*. 5900 cal. yr BP, and then reached the Guanzhong Basin at *c*. 5600 cal. yr BP [54] and the Xishanping site *c*. 5000 cal. yr BP [55] (Figure 1). Consequently, these findings draw a temporal-spatial route for the northward spread of rice cultivation, and also have extensive implications to add to the discussion of rice domestication [14], [56], [57].

Phytolith results together with numerous agricultural tools, i.e., denticulate sickles, adzes, slabs (metate, *mo-pan*), mullers (*mo-bang*), and others, recovered from the Tanghu site suggest that millet and rice farming could be traced back to 7800 cal. yr BP in the Middle Yellow River region [26], [58]. Together with the crop complex evidence from the Yuezhuang site [11], these findings suggest that the source of mixed millet-rice cultivation may be located in the Mid-Lower Yellow River region 8000 years ago. The adoption of millet-rice cultivation would significantly change the proportion of food production in the Neolithic subsistence economy that eventually led to the formation of a continuous Ancient Yellow River Civilization.

### Conclusions

Our evidence indicates that broomcorn millet was cultivated in this region 7800 cal. yr BP during the Peiligang Culture. Rice exploitation took place in the period from 7800 to 4500 cal. yr BP. Our data provide new evidence of mixed broomcorn millet and rice farming record in the Peiligang Culture in the Middle Yellow River region. The findings have implications for understanding broomcorn millet domestication, northward spread of rice cultivation, and the formation of mixed millet-rice farming, thus the formation and continuance of the Ancient Yellow River Civilization.

It should be pointed out that more work is needed to complete the picture of primitive agriculture at the Tanghu site in the Peiligang Culture due to the limited crop phytoliths found for this study. Well-dated multi-proxy data on pollen, starch analysis, and plant macrofossils are needed in future studies.
